# Supplementary material for: Computer-assisted initial diagnosis of rare diseases
Source: PeerJ. 2016 Jul 21;4:e2211. doi: 10.7717/peerj.2211 (PMC4963223; doi:10.7717/peerj.2211)
Supplement: Appendix S1 — Additional Methods and Results for Efficient Computer-Assisted Diagnosis of Rare Diseases Using Rare Disease Discovery [file peerj-04-2211-s001.pdf]

Supporting Appendix 1 containing:

Supporting Methods and Results – Text with supplementary methods and results (pages 3-9).

Supporting Figure 1 – Two examples of RAMEDIS report cards (page 10).

Supporting Figure 2 – Number of diseases to be considered vs. presence of the correct disease in that list for the retrospective study of previously diagnosed rare disease patients (page 10).

Supporting Figure 3 – Estimating the probability that a score $DS_i \leq k$ might be obtained from a random set of symptoms (page 11).

Supporting Figure 4 – Effect of unreported symptoms on the maximum DS_i scores for patients (page 11).

Supporting Figure 5 – Effect of the evolution in the ORPHANET dataset on the F1-Score of RDD (page 12).

Supporting Figure 6 – Effect of the evolution in the ORPHANET dataset on the effect of unreported symptoms on the maximum DSi scores for patients (page 13).

Supporting Table 1 – Symptoms used to perform the experiments summarized in Tables 1 and 2 (page 15).

Supporting Table 2 – Estimating the probability that a difference between scores $\Delta DS_i < x$ is significant at three p-value levels, when a varying number of symptoms is submitted to RDD (page 16).

Supporting Appendix 1: Additional Methods and Results for Computer-Assisted Initial Diagnosis of Rare Diseases

Rui Alves[1,*,§], Marc Piñol, [2,*], Jordi Vilaplana[2], Ivan Teixido[2], Joaquim Cruz[1], Jorge Comas[1], Ester Vilaprinyo[1], Albert Sorribas[1], Francesc Solsona[2,§],

[1]Departament d'Informàtica i Enginyeria Industrial, Universitat de Lleida, Av. Jaume II nº 69, 25001 LLeida, Spain.

[2]Departament de Ciències Mèdiques Bàsiques & IRBLleida, Universitat de Lleida, Montserrat Roig nº 2, 25008 LLeida, Spain.

e-mails: wrrzag666@gmail.com
jordi@diei.udl.cat
iteixido@diei.udl.cat
joaquimcruz92@gmail.com
jorgecomasp@gmail.com
evilaprinyo@cmb.udl.cat
albert.sorribas@cmb.udl.cat
francesc@diei.udl.cat
ralves@cmb.udl.cat

*These two authors contribute equally for the study

§Corresponding authors:

Francesc Solsona: Departament d'Informàtica i Enginyeria Industrial, Universitat de Lleida, Av. Jaume II nº 69,
25001 LLeida, Spain.
email: francesc@diei.udl.cat
tel: 00 34 973 70 27 35
fax: 00 34 973 70 27 02

Rui Alves: Departament de Ciències Mèdiques Bàsiques & IRBLleida, Universitat de Lleida,
Edifici de Recerca Biomèdica I, Av. Rovira Roure 80, 25198 LLeida, Spain.
email: ralves@cmb.udl.cat
tel: 00 34 973 70 24 25
fax: 00 34 973 70 24 26

TECHNOLOGY UNDERLYING THE RARE DISEASE DISCOVERY PROTOTYPE

The technology behind the web application is the GRAILS framework, a web application framework built for the Java Virtual Machine that uses the Groovy programming language. GRAILS uses a MVC (Model View Controller) pattern that allows for a full integration between the model (and the database) and the view (user interface). With built-in database access and modeling, it enables easy abstraction and decoupling between these two parts of the application, permitting easy database migrations. This also helps hiding database complexity and access to information in an object-oriented way. JQuery and Ajax were also used in order to provide dynamic web capabilities like autocomplete. A powerful front-end framework for faster and easier web development (Twitter Bootstrap) was included, streamlining the styling and design of the web interface. Built for the JVM, this framework also enables easy integration with Java packages, plugins and wrappers.

The database design provides a welcome positive side-effect: it is trivial to keep the database up-to-date. A periodic download of the ORPHANET data every three months, followed by upload of that data to our database can be done in minutes, facilitating that RDD is kept up to date and usable over the long run. Currently, the database has a total of 6 915 diseases and 2 110 symptoms. There is a total of 101 840 records representing relations between symptoms and diseases.

We note that the symptoms-disease association file from the ORPHANET dataset are re-curated by us in order to ensure that the automated processing of the xml file made available by ORPHANET is done without mistakes. Although it does not always happen, in some versions of the xml files we downloaded had one or more tags that were not properly closed. In addition, in the earlier versions of the files, the symptoms had not yet been fully converted to their synonymous terms in

the HPO (Human Phenotype Ontology)[1]. We performed a script analysis to identify those terms that were not in HPO and transformed them into their HPO synonyms. In the last 3 versions of the ORPHANET xml file, we found that HPO nomenclature has been fully implemented.

CHOOSING THE APPROPRIATE PREDICTION METHODS

Other classification approaches to predicting rare diseases based on symptoms were tested. First, we tested an additional ranking function that takes into consideration how frequently each symptom is thought to be associated with the disease. This information is provided in the ORPHANET dataset, which associates qualitative frequency information to a symptom, when it is associated to a disease (Very Frequent, Frequent, and Occasional). This tested function as the form:

$$DS_i' = 1 - \frac{\sum_{j=1}^{n} \delta_j}{Max[S_{user}, S_{Disease\ i}]} \qquad \text{Eq. A1}$$

In Eq. A1 S_{user} represents the number of symptoms provided by the user, $S_{Disease\ i}$ represents the number of symptoms of disease *i* stored in the database, and $Max[S_{user}, S_{Disease\ i}]$ represents the largest number between S_{user} and $S_{Disease\ i}$. *n* represents the number of symptoms that are different between the set submitted by the user and the set associated to any given rare disease in the database. δ_j measures the qualitative frequency at which symptom *j* has been found to associate to disease *i* in the past (see above). Given that there were only three categorical frequency associations (Very Frequent, Frequent, Occasional), δ_j was considered to have one of three values. $\delta_j = 1$ if the symptom is either very frequently associated to the disease *i* or is a symptom that is provided by the user; $\delta_j = 0.75$ if the symptom is frequently associated to the disease *i;* finally, if the symptom is only occasionally associated to disease *i*, $\delta_j = 0.5$. It can be shown that ,

$-1 \leq DS_i \leq DS_i' \leq 1$. However, even though $DS_i' \neq DS_i$, the list of diseases is ranked in the same order by both scores (data not shown). Because more calculations are required to estimate DS_i', using this score for ranking leads to slower computation. Hence, we discarded DS_i'.

Second, we also trained and tested algorithms based on Support Vector Machines, Neural Networks, Bayesian Networks, Random Trees, and Random Forests. Invariably, these algorithms required extensive training and prediction time, and their best performance was always about one order of magnitude lower than that of the algorithm and score described in this paper. They were also orders of magnitude slower in predicting the disease and required more computational resources for doing so.

RETROSPECTIVE STUDY OF PREVIOUSLY DIAGNOSED RARE DISEASE PATIENTS

RAMEDIS is a server that provides management services for medical doctors diagnosing, treating and managing rare disease patients. Its database contains short report cards with at most 3 sentences about 1099 patients with confirmed rare disease clinical diagnostics.

The information for about 60% of these patients is public, although anonymized. From these approximately six hundred patients, nearly half have metabolic rare diseases that were diagnosed in screening programs at a preclinical stage. Of the remainder three hundred patients, one hundred and eighty seven had a confirmed clinical diagnosis associated with a report card that described at least one symptom. Examples of the procedure are given

We took these 187 patients and reconstructed their symptoms from the individual report cards. In some cases this is easy, and report cards were very clear (for example: patient with seizures or hypotonia). In other cases the symptoms were vaguely described and hard to reconstruct. For example, “hearing problems” or “hearing loss” could be any of the following: “Conductive deafness/hearing loss”, “Central deafness/hearing loss”, “Sensorineural

deafness/hearing loss", or "Hearing loss/hypoacusia/deafness". Another example, "Infection" could be any of the following: "Immunodeficiency/increased susceptibility to infections/recurrent infections", "Recurrent urinary infections", "Chronic skin infection/ulcerations/ulcers/cancrum", or "Repeat respiratory infections". In these cases, we opted for including all possibilities rather than eliminating the symptom. This decision was made because eliminating the symptom would have meant discarding additional patients from an already small set, as all reported symptoms were often ambiguous. An example of two report cards and their processing is shown in Supporting Figure 1. The patients, their symptoms, and their clinically confirmed diagnosis can be manually accessed and compiled from the RAMEDIS website. Supporting Figure 2 plots the accumulated frequency of the score for the correct (and best) prediction.

BENCHMARKING THE RARE DISEASE DISCOVERY PROTOTYPE

The rare disease prediction algorithm was extensively benchmarked to evaluate the effect of absent and unrelated symptoms on diagnostic precision. In addition, we also tested how the changes in the ORPHANET dataset could affect the results. These benchmarks relied on several sets of tests, all run using Stochastic Monte-Carlo simulations.

Aggregated effects of unreported and unrelated symptoms on prediction accuracy of the Rare Disease Discovery Algorithm

The first benchmark test was done by generating several random sets of 10 000 patients, each with all the symptoms associated to a specific but randomly chosen rare disease. Then, for increasing percentages of the patients in a given random set either 1, 2, 3, 4, 5, 10, or 20 symptoms were randomly added or deleted to create noise. Then, the noisy sets of symptoms were used by the RDD algorithm to predict the rare disease that generated them. The precision *p*,

sensitivity *s*, and *F-Score* of the RDD prediction algorithm were calculated for each set of patients. The results are summarized in Figure 2 of the main text and discussed in the main manuscript.

Effects of unreported symptoms on prediction accuracy of the Rare Disease Discovery Algorithm

The second benchmark test was done by again generating several random sets of 10 000 patients, each with all the symptoms associated to a specific but randomly chosen rare disease. Then, for increasing percentages of the patients in a given random set, either 25%, 50%, or 75% of the symptoms were deleted to create noise. Finally, the noisy sets of symptoms were used by the RDD algorithm to predict the rare disease that generated them. These simulations represent situations where not all symptoms are known to the user during diagnosis. The precision *p*, sensitivity *s*, and *F-Score* of the RDD prediction algorithm were calculated for each set of patients. The results are summarized in Figure 3 of the main text and discussed in the main manuscript.

Estimating significance for DS_i scores and testing the performance of RDD in misdiagnosing patients that do not suffer from rare diseases

It is important to estimate how large DS_i must be for a user to be sure that the set of symptoms being submitted to RDD (Rare Disease Discovery) are not the result of randomly associated symptoms. A third benchmark of the RDD algorithm was done to estimate this DS_i value. This estimation was done in following way. Consider that there are 13 698 diseases and 2 528 symptoms in our database. The average number of symptoms associated to a disease is 42, with a standard deviation of 59. To calculate the probability that a given DS_i for a set of symptoms produced by a user is statistically significant we generated 10 000 random vectors of symptoms. The population of the 10 000 vectors had an average number of symptoms equal to 42, with a standard deviation of 59. Given that these vectors were random, by plotting $f = (1 - Accumulated\ frequency\ of\ DS_i)$ as a function of DS_i (Supporting Figure 3) we are able to

estimate the probability that a given score is achieved simply by choosing a random combination of symptoms. This experiment estimates that a score $DS_i \geq 0.5$ has a probability lower than 0.0001 of being obtained by choosing a random set of symptoms. If we lower the probability to 0.01, then $DS_i \geq 0.25$. In fact, the median DS_i score for a random choice of symptoms is less than 0.01.

Estimating significance levels for the differences between two ds_i scores

In the previous section we describe an experiment that allowed us to estimate that if DS_i>0.5, one can be 99.99% sure that the score was not obtained by choosing a random set of symptoms.

Another issue is that of determining how significant are the differences between two DS_i scores for the same set of symptoms. Estimating this is much more complicated because the significance will depend on the number of symptoms one submits for the prediction. A final benchmark experiment was done in order to provide a best scenario estimation for how statistically significant the differences between two DS_i scores are.

In this fourth and final benchmark we performed the following Monte Carlo simulation experiments. For each disease we created all possible sets of k symptoms, where *k*=1, 2, 3, 4, 5, 10, 20, and 50 symptoms that are associated to that disease (taking care to eliminate diseases in the simulation that had less than the simulated number of symptoms). Then, for each *k*, we calculated DS_i for the correct disease. We call this list $DS_{i\ correct}$ In parallel, for each *k* and for each set of symptoms, we calculated DS_i for all diseases that were not the one from which we had extracted the set of symptoms. We call this list $DS_{i\ incorrect}$.

Then, for each k we created a list ΔDS_i, where each element of the list corresponds is obtained by subtracting quantile j of $DS_{i\ incorrect}$ from quantile j of $DS_{i\ correct}$. The results are presented in Supporting Table 2 and interpreted in the following way. For the same number of

submitted symptoms, in the context of the disease-symptoms association matrix, the differences between corresponding quantiles of the $DS_{i\ \mathrm{correct}}$ and $DS_{i\ \mathrm{incorrect}}$ lists provide a proxy to evaluate how different two DS_i scores (one correct and one incorrect) must be for that difference to be significant. Thus, if users submit for example one symptom and want a certainty of 99.9% that two DS_i scores are different, Supporting Table 2 tells us that the two scores should differ by at least 0.14. How can this be interpreted? For example, the difference between the score for the most highly ranked disease and that for the second best guess by RDD needs to differ by at least 0.14, if one want to state that the prediction is significantly ($p<0.001$) better than the second best guess.

It is important to benchmark the performance of RDD with patients that have symptom(s) associated to rare diseases, without suffering from those diseases. This is a very real scenario, as many of the symptoms are common between rare and non-rare diseases. A possible test would be to create synthetic patients from other diseases, adding random rare disease symptoms and running RDD. However, we note that RDD only allows users to choose symptoms that have been previously associated to at least one rare disease. Hence, testing RDD's performance with synthetic patients from non-rare diseases is formally equivalent to generating synthetic patients with random associations of rare-disease symptoms. This is the same test that was done to determine significance for DS_i scores. In other words, only when DS_i is larger than 0.5, does RDD ensure that the patient has a rare disease, with a probability higher than 0.9999.

Accurate predictions in the absence of statistically significant DS_i scores

Taken together, the four benchmark experiments described in the main manuscript show that DS_i decreases sharply with noise; however, even if DS_i is below the statistically significant level, it can still be used to accurately predict the correct rare disease, although with a lower confidence (see above). For example, in Supporting Figure 4 we show Box plots of the maximum DS_i scores for all patients in the second benchmark test. We see that when patients have 50% absent symptoms,

the maximum score is still almost always above 0.5, which is the 0.0001 significance level determined in benchmark 3. Only when 75% of the symptoms are absent do we get maximum DS_i scores that are equal to or lower than 0.5 for more than 50% of the patients.

Effect of evolving datasets: ORPHANET dataset of December 2014 vs. ORPHANET dataset of December 2015

Given that the dataset we used is annotated by humans and evolves, we wanted to have an estimate of how much the changes might affect the predictive capabilities of RDD. To achieve this we repeated the tests described in all the previous subsections of "BENCHMARKING THE RARE DISEASE DISCOVERY ALGORITHM" for the ORPHANET dataset of 2015. What we found was that the difference in F-Score of RDD between the two sets was smaller than 3% when noise was large (20 noisy symptoms) and less than 0.2% when symptoms were accurate (Supporting Figure 5). We also observe that the median score of the correct prediction when 25%, 50%, or 75% of symptoms are absent increased by approximately 20% when we changed the 2014 dataset for the 2015 dataset (Supporting Figure 6). These results suggest that the human curation of the ORPHANET dataset is improving over time, which also improves the quality of the results of computer assisted DDX tools that use them, as is the case of RDD.

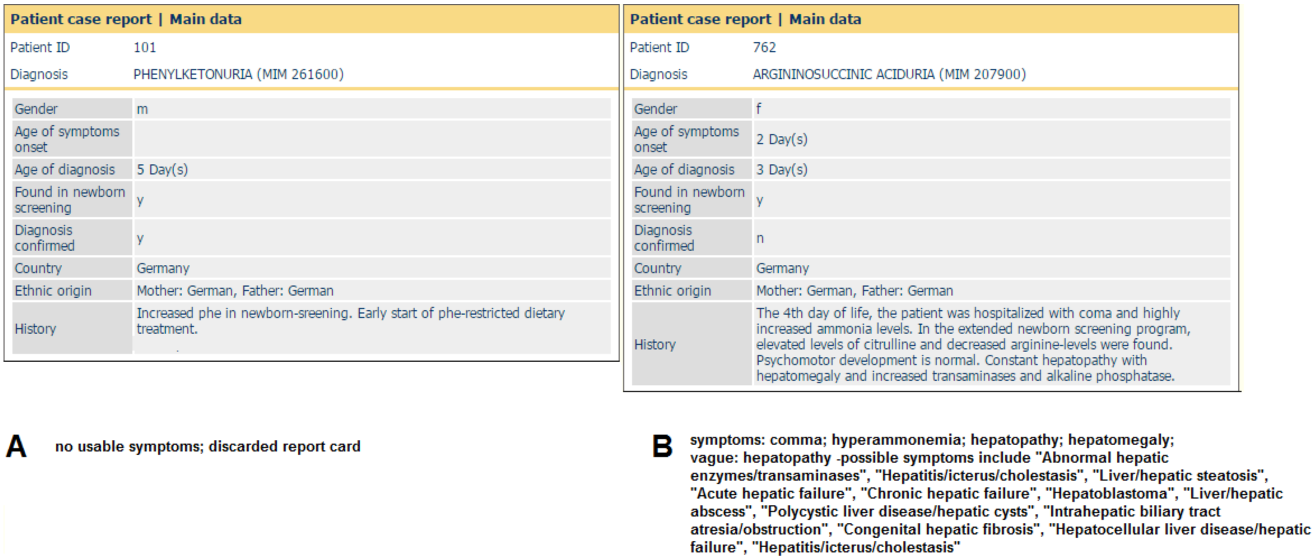

Supporting Figure 1 – Two examples of RAMEDIS report cards. **A**: Example of a report card that could not be used, as no symptoms were reported. **B**: Example of a report card that could be used, but had vague description of some symptoms.

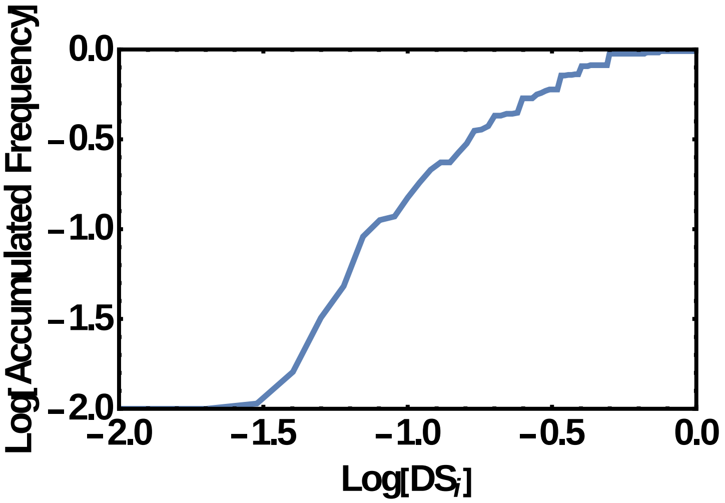

Supporting Figure 2 – Cumulative frequency of the highest score for the retrospective study of previously diagnosed rare disease patients.

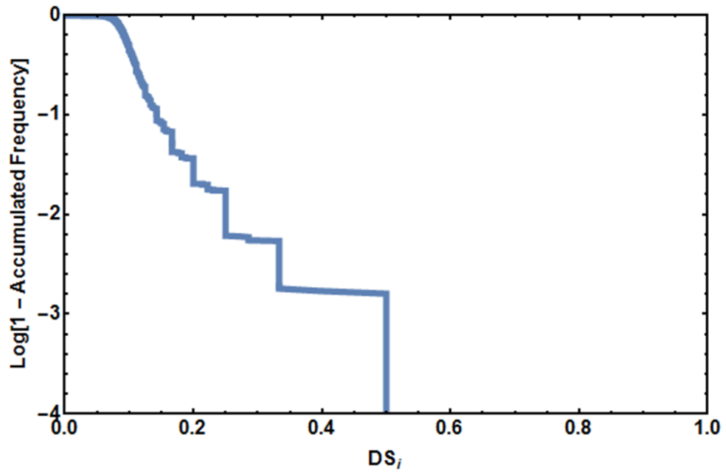

Supporting Figure 3 – Estimating the probability that a score DS_i≤k might be obtained from a random set of symptoms. The score DS_i is represented in the x-axis, while the logarithm of 1 – the accumulated frequency of DS_i is represented in the y-axis. DS_i score values higher than 0.5 have a probability of 0.0001 of being obtained from a random set of symptoms.

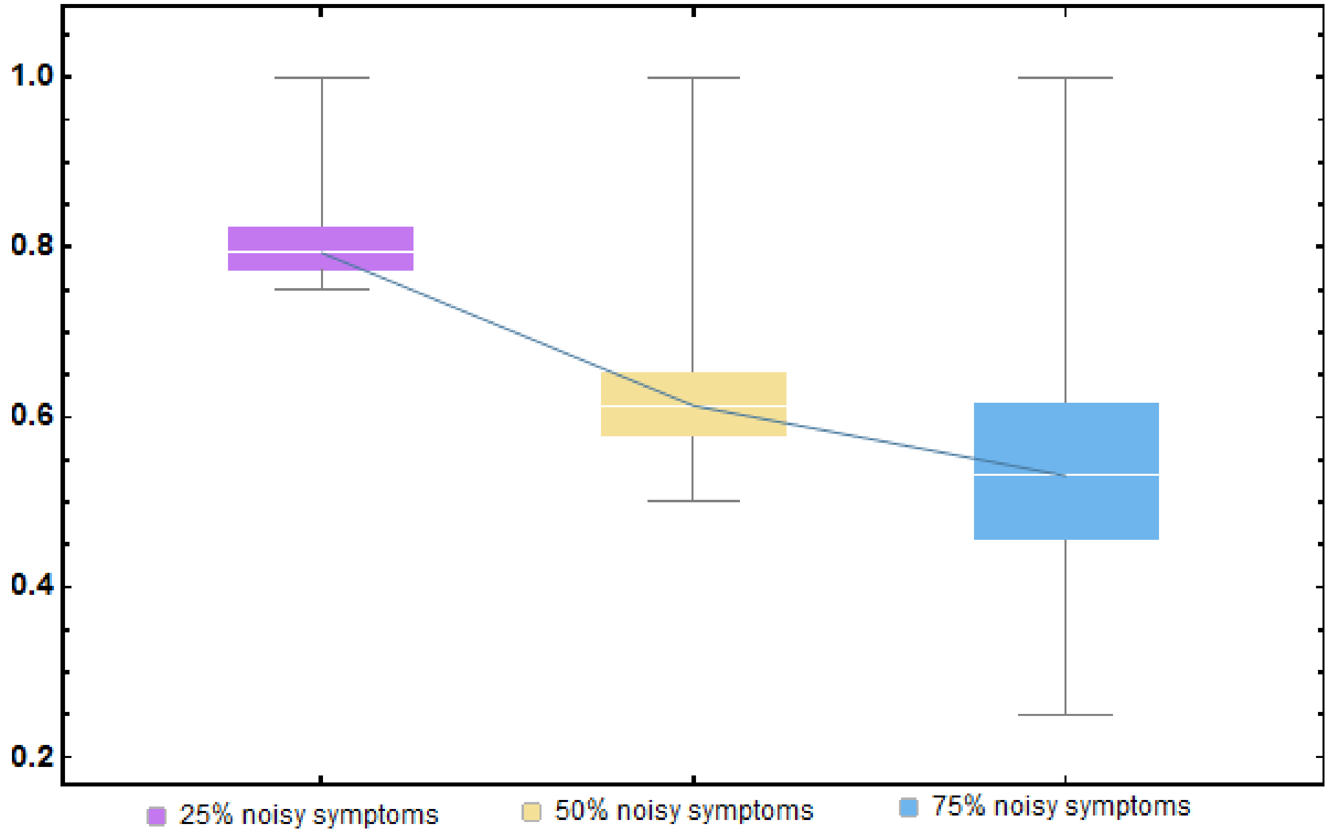

Supporting Figure 4 – Effect of unreported symptoms on the maximum DS_i scores for patients. Here we present Box plots of the maximum DS_i scores for all patients when 25%, 50%, or 75% of the symptoms are absent. The median maximum scores are joined by a blue line. The boxes indicate the 0.25 and 0.75 quartiles in each dataset.

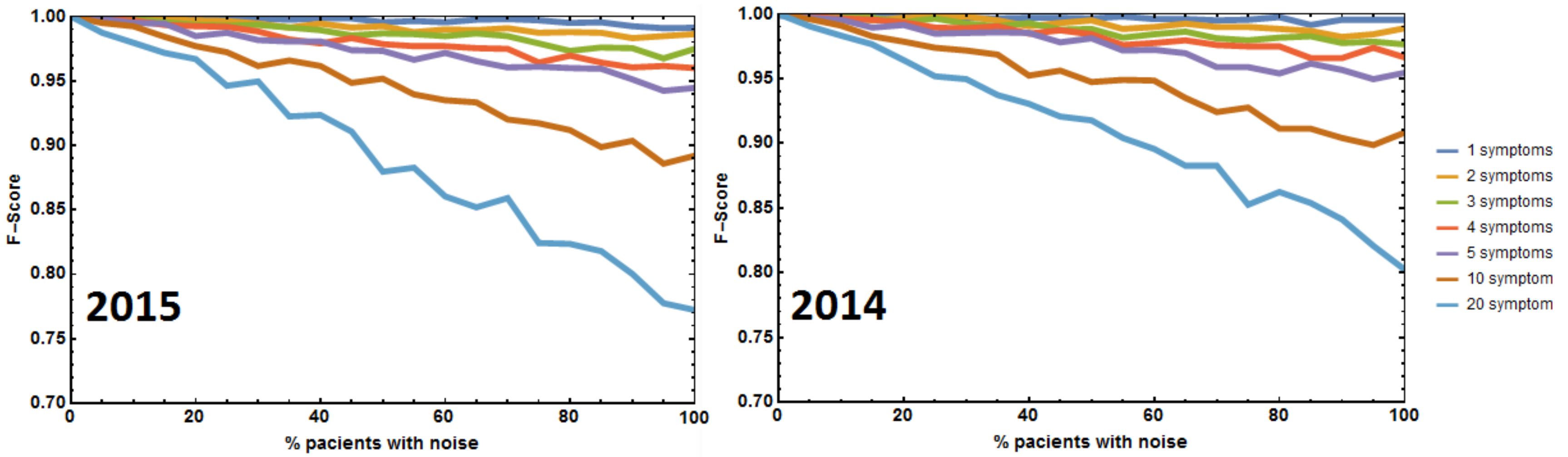

Supporting Figure 5 – Effect of the evolution in the ORPHANET dataset on the F1-Score of RDD. Comparison of the datasets from December 2014 and 2015. The effect of the dataset from different years is less than 3%.

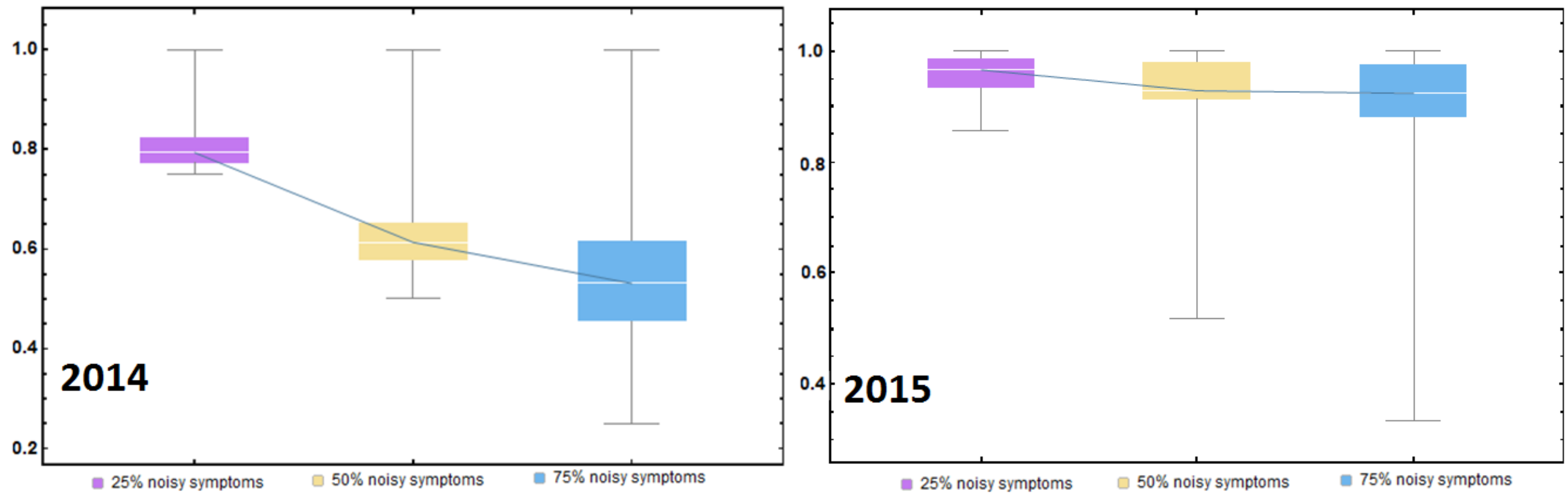

Supporting Figure 6 – Effect of the evolution in the ORPHANET dataset on the effect of unreported symptoms on the maximum DS_i scores for patients. Comparison of the datasets from December 2014 and 2015. The median maximum scores are joined by a blue line. The boxes indicate the 0.25 and 0.75 quartiles in each dataset. The median scores of the newest dataset are higher than those of the 2014 dataset, indicating an improvement in the quality of the RDD predictions when symptoms are under-reported.

Supporting Table 1 – Symptoms used to perform the experiments summarized in Tables 1 and 2.

Disease	Initial Symptom	Rank at First symptom	Additonal symptoms required for the appropriate disease to be ranked as 1st prediction
**Beta-Thalassemia**	Chronic skin infection/ulcerations/ulcers/cancrum	67th	Humour troubles/anxiety/depression/apathy/euphoria/irritability<br>Anaemia
**Canavan Disease**	Motor deficit/trouble	23rd	Seizures/epilepsy/absences/spasms/status epilepticus<br>Retinitis pigmentosa/retinal pigmentary changes<br>Hypotonia<br>Contractures/cramps/trismus/tetania/claudication/opisthotonos
**Down Syndrome**	Strabismus/squint	244th	Sterility/hypofertility<br>Microstomia/little mouth<br>Insulin-independent/type 2 diabetes
**Fabry Disease**	Renal failure	111th	Anorexia<br>Humour troubles/anxiety/depression/apathy/euphoria/irritability<br>Renal disease/nephropathy<br>Nausea/vomiting/regurgitation/merycism/hyperemesis<br>Myalgia/muscular pain<br>Thick lips<br>X-linked recessive inheritance
**Goldblatt Syndrome**	Hip dislocation/dysplasia/coxa valga/coxa vara/coxa plana	81st	Delayed dentition/eruption of teeth/lack of eruption of teeth<br>Respiratory distress/dyspnea/respiratory failure/lung volume reduction
**Turner Syndrome**	Pigmented naevi/naevus pigmentosus/lentigo	21st	Thin/hypoplastic toe nails
**Uncombable Hair Syndrome**	Albinism (hair)	1st	Albinism (hair)
**Williams Syndrome**	Renal failure	121st	Angor pectoris/myocardial infarction<br>Thin/hypoplastic toenails<br>Late puberty/hypogonadism/hypogenitalism<br>Osteosclerosis/osteopetrosis/bone condensation
**Yunis-Varon Syndrome**	Sternal/sternum anomalies	7th	Cardiomyopathy/hypertrophic/dilated<br>Poorly ossified skull/calvarium<br>Absent/small toenails/anonychia of feet<br>Blepharophimosis/short palpebral fissures<br>Absent/small fingernails/anonychia of hands<br>Anteverted nares/nostrils<br>Hip dislocation/dysplasia/coxa valga/coxa vara/coxa plana<br>Hypotonia
**Zellweger-like Syndrome without Peroxisomal Anomalies**	High forehead	31st	Broad nasal root<br>Expressionless face/amimia

Supporting Table 2 – Estimating the probability that a difference between scores $\Delta DS_i < x$ is significant at three p-value levels, when a varying number of symptoms is submitted to RDD.

Number of symptoms	$\Delta DS_i\left(p-value<0.01\right)$	$\Delta DS_i\left(p-value<0.005\right)$	$\Delta DS_i\left(p-value<0.001\right)$
1	$\Delta DS_i \le 0.01$	$\Delta DS_i \le 0.025$	$\Delta DS_i \le 0.14$
2	$\Delta DS_i \le 0.001$	$\Delta DS_i \le 0.005$	$\Delta DS_i \le 0.015$
3	$\Delta DS_i \le 0.001$	$\Delta DS_i \le 0.001$	$\Delta DS_i \le 0.010$
4	$\Delta DS_i \le 0.001$	$\Delta DS_i \le 0.001$	$\Delta DS_i \le 0.007$
5	$\Delta DS_i \le 0.001$	$\Delta DS_i \le 0.001$	$\Delta DS_i \le 0.005$
10	$\Delta DS_i \le 0.001$	$\Delta DS_i \le 0.001$	$\Delta DS_i \le 0.001$
20	$\Delta DS_i \le 0.001$	$\Delta DS_i \le 0.001$	$\Delta DS_i \le 0.001$
50	$\Delta DS_i \le 0.001$	$\Delta DS_i \le 0.001$	$\Delta DS_i \le 0.001$
